# Appraisal of Systemic Treatment Strategies in Early HER2-Positive Breast Cancer—A Literature Review

**DOI:** 10.3390/cancers15174336

**Published:** 2023-08-30

**Authors:** Danilo Giffoni de Mello Morais Mata, Rania Chehade, Malek B. Hannouf, Jacques Raphael, Phillip Blanchette, Abdullah Al-Humiqani, Monali Ray

**Affiliations:** 1 Division of Medical Oncology, London Regional Cancer Program, London Health Sciences Centre, Western University, London, ON N6A 5W9, Canada; jacques.raphael@lhsc.on.ca (J.R.); phillip.blanchette@lhsc.on.ca (P.B.); 2 Division of Medical Oncology, Odette Cancer Centre, Sunnybrook Health Sciences Centre, University of Toronto, Toronto, ON M4N 3M5, Canada; rania.chehade@sunnybrook.ca (R.C.); abdullah.al-humiqani@sunnybrook.ca (A.A.-H.); 3 Department of Internal Medicine, Western University, London, ON N6A 3K7, Canada; malek.hannouf@lhsc.on.ca; 4 Division of Medical Oncology, Markham Stouffville Hospital, Markham, ON L3P 7P3, Canada; moray@oakvalleyhealth.ca

**Keywords:** invasive breast carcinoma, human epidermal growth factor receptor 2-positive, trastuzumab, pertuzumab, monoclonal antibody, chemotherapy, neoadjuvant and adjuvant treatment

## Abstract

**Simple Summary:**

HER2-positive breast cancer (BC) is associated with an aggressive clinicopathological nature and is known to have a poor prognosis. Their tumor proliferation features can lead to visceral metastasis disseminating to the brain, liver, lung, and bone. Delivering standard chemotherapy HER2 blockers is strongly associated with better outcomes and can help increase the suitability for breast-conserving surgery. It is crucial to identify patients who should be selected for systemic cancer treatment before or after surgery and to decide the most appropriate option in each case. A thorough search and data collection were carried out using electronic scientific libraries and compiled with the most relevant and updated information about the stratification of BC recurrence risk and standard treatment options for each case.

**Abstract:**

Background: The overexpression of the human epidermal growth factor receptor 2 (HER2+) accounts for 15–20% of all breast cancer phenotypes. Even after the completion of the standard combination of chemotherapy and trastuzumab, relapse events occur in approximately 15% of cases. The neoadjuvant approach has multiple benefits that include the potential to downgrade staging and convert previously unresectable tumors to operable tumors. In addition, achieving a pathologic complete response (pCR) following preoperative systemic treatment is prognostic of enhanced survival outcomes. Thus, optimal evaluation among the suitable strategies is crucial in deciding which patients should be selected for the neoadjuvant approach. Methods: A literature search was conducted in the Embase, Medline, and Cochrane electronic libraries. Conclusion: The evaluation of tumor and LN staging and, hence, stratifying BC recurrence risk are decisive factors in guiding clinicians to optimize treatment decisions between the neoadjuvant versus adjuvant approaches. For each individual case, it is important to consider the most likely postsurgical outcome, since, if the patient does not obtain pCR following neoadjuvant treatment, they are eligible for adjuvant T-DM1 in the case of residual disease. This review of HER2-positive female BC outlines suitable neoadjuvant and adjuvant systemic treatment strategies for guiding clinical decision making around the selection of an appropriate therapy.

## 1. Introduction

Breast cancer (BC) is a heterogenous disease with a wide range of tumor morphology phenotypes [1]. Nearly 75% of BC cases demonstrate luminal differentiation expressing endocrine receptor (ER) positivity [2]. The overexpression of the human epidermal growth factor receptor 2 (HER2+), determined by immunohistochemistry (IHC) and in situ hybridization (ISH) methods, accounts for 15–20% of all BC cases [3].

ER+ HER2– BC has the most indolent course and the best prognosis among the BC subtypes, despite the tropism for disseminating to bones [4,5]. Conversely, HER2+ BC is associated with an aggressive clinicopathological nature and a poor prognosis. Their tumor proliferation features can lead to visceral metastasis disseminating to the brain, liver, and lung, in addition to the bone [6]. Eventually, most patients who have HER2+ BC recurrence will die of their disease [7]. While most patients with luminal A, ER+ HER− BC, are treated in the adjuvant setting with oral endocrine therapy and do not require chemotherapy, the mainstay of treatment for the luminal B, ER+ HER2+ BC subtype is chemotherapy with the monoclonal antibody (mAB) trastuzumab, administered before or after surgery [5,8].

The neoadjuvant approach has multiple benefits that include the potential to improve staging, converting previously unresectable tumors to operable tumors, and, from a cosmetic perspective, an increased suitability for breast-conserving surgery. Exposing patients to neoadjuvant chemotherapy (NAC) can predict the chemosensitivity of tumors to conventional cytotoxic agents and, most importantly, is prognostic of the outcome, helping to optimize treatment decisions in patients with residual cancer after chemotherapy [9].

The advent of HER2-targeted therapies has led to a novel systematic approach, revolutionizing the treatment of HER2+-enriched tumors and improving the prognosis of this particular BC population [6]. Between 47% and 65.2% of patients receiving NAC plus trastuzumab will successfully achieve a complete eradication of malignant cells at the microscopic level, also known as pathological complete response (pCR) [10,11,12]. Many studies have demonstrated that achieving a pCR, following preoperative chemotherapy, is strongly associated with better outcomes [13,14]. The CTNeoBC pooled analysis of 12 trials including approximately 12,000 BC patients treated with preoperative chemotherapy followed by surgery showed that pCR, which included the breast and lymph node (LN), was associated with improved event-free survival (EFS) and OS when compared to pCR in the breast alone [9]. The tumor response to NAC varies depending on the BC subtype. There is a high association between pCR and improved survival in the HER2+ BC, as well as in the triple-negative breast cancer (TNBC) subtype, but it is more significant in latter group, despite HER2+ BC being a candidate for targeted treatment with trastuzumab [9]. Among the HER2+ subgroups, ER− is more susceptible to achieving pCR events as compared to the luminal B, ER+ phenotype [9,15].

Although two decades have passed and trastuzumab remains the cornerstone therapy for the HER2 populations, relapse events occur in approximately 15% of BC cases, even beyond the completion of standard chemotherapy with trastuzumab [4,16]. Out of these, 8% will reoccur within 4 years, despite the improvement in pCR rates [17,18]. Much work is needed to better understand HER2+ BC, especially as research indicates that acquired resistance may be due to the immunogenic nature of the pathophysiology. Further research will also help develop more effective therapies to counteract this acquired resistance [19].

This article highlights the latest evidence of neoadjuvant and adjuvant treatments for patients diagnosed with HER2+ BC and includes the following: chemotherapy with or without anthracycline, single or dual blockage agents against HER2, and an optimal therapy duration. In addition, this paper will briefly review critical oncodriver mutations and molecular pathways crossing with HER2+, as well as inherent caveats, pitfalls, and future directions.

## 2. Materials and Methods

To warrant the inclusion of updated medicine-based evidence relevant to clinical practice, a literature search was conducted with scientific journals of published systematic reviews and meta-analyses, with clinical trials and abstracts from oncology conferences, and by searching the Embase, Medline, and the Cochrane electronic libraries. The search was conducted between January 2002 and May 2023 in the English language.

## 3. HER2 as an Oncogenic Molecular Driver

HER2 is an oncogenic membrane-spanning receptor of a signaling pathway located in the intracellular and extracellular domains (ECD). HER2 belongs to the family of epidermal growth factor receptor tyrosine kinases known as EGFR or ErbB, encompassing four structurally analogous receptors including HER1, HER2, HER3, and HER4 [20,21]. When HER2 is overexpressed, it generates a rearrangement of the HER family of dimers, leading to a significant augmentation of the HER2–containing heterodimers and homodimers and enabling the HER2 receptor activation by dimerization with another HER family member [22,23]. Hence, by eliciting a cascade of transduction signals partaking in the PI3K/Akt/mTOR and MAPK molecular alleyways, it leads to the regulation of cellular proliferation and differentiation, thus triggering tumor growth [23].

## 4. Classification of HER2+ Tumors

Based on the American Society of Clinical Oncology and the College of American Pathologists (ASCO/CAP) guidelines, HER2+ is defined as IHC 3+ (circumferential membrane staining including >10% of cells) or IHC 2+ combined with HER2 amplification by an ISH assay of the invasive component of a BC specimen using single/dual-probe ISH. There are intricacies for determining HER2 positivity based on the ISH pattern. Nonetheless, to summarize, a single signal probe ISH is based on the average HER2 copy number and is considered negative when the average HER2 copy number is <4.0 signals/cell and positive when the average HER2 copy number is ≥6.0 signals/cell. However, when the estimated HER2 is ≥4.0 and <6.0 signals/cell, the HER2/chromosome enumeration probe 17 (CEP17) ratio should be ≥2.0 in order for HER2 to be considered positive [24].

## 5. Treatment Strategies for HER2+ Breast Cancer

### 5.1. Monoclonal Antibody against HER2

Trastuzumab was the first targeted humanized mAB against HER2 to be approved by the US Food and Drug Administration (FDA) [25,26]. By binding to domain-4 on the juxtamembrane region of the HER2 ECD, trastuzumab disrupts ligand-independent downstream signaling pathways to the intracellular environment, triggering antibody-dependent cell-mediated cytotoxicity. Through immune mechanisms, where antibody-coated cells target the surface of tumor-derived antigens, cascade processes are prompted to inhibit cell cycle arrest and tumor angiogenesis via antibody-dependent cellular toxicity (ADCC) [27,28].

**Figure 1:**
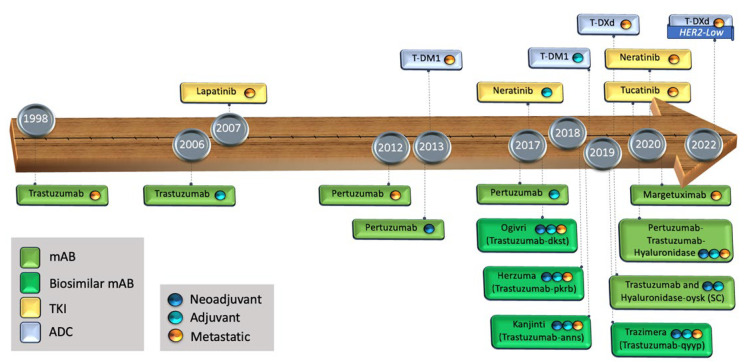
Timeline of the FDA-approved targeted treatments against the HER domains. mAB: monoclonal antibody, TKI: tyrosine kinase inhibitor, ADC: antibody drug conjugate, SC: sub-cutaneous.

The advent of trastuzumab has been a remarkable breakthrough in the history of BC, due to its ability to prolong survival and improve quality of life (Figure 1). Trastuzumab has dramatically changed the trajectory of HER2+ populations, not only in BC but in other malignancies such as those involving the gastro-esophageal junction, colorectal, and endometrial carcinomas [29,30,31,32].

The first usage of trastuzumab was in combination with conventional chemotherapy in metastatic HER+ BC [33,34]. These outcomes were further replicated in non-metastatic HER+ disease [35,36]. In this manner, trastuzumab, in combination with taxane-based chemotherapy, has paved the way for understanding multimodal treatments in the adjuvant and neoadjuvant settings in HER2+ BC [35,37].

A study conducted by The Early Breast Cancer Trialists’ Collaborative group (EBCTCG), with 13,864 patients revealed noteworthy results when adding trastuzumab to standard adjuvant chemotherapy. At 10 years, there was an absolute risk reduction in breast cancer-specific mortality (BCSM) by 6.4% (95% CI, 4.9–7.8) and in all-cause mortality by 6.5% (95% CI, 5.0–8.0). The 10-year relapse event rate decreased by 9% (95% CI, 7.4–10.7) when compared to no trastuzumab. The risk of recurrence was more pronounced in the early years, as compared to 10 years and beyond [36].

### 5.2. Dual HER2 Blockage with mABs

Pertuzumab is a recombinant humanized mAB binding to subdomain II of the ECD of HER2, inhibiting dimerization and subsequent receptor ligand-dependent signaling [3]. In contrast to trastuzumab, pertuzumab interrupts the HER heterodimerization on the HER1 (or EGFR), HER2, HER3, and HER4 domains, stopping the molecular transduction of downstream tumor signaling [38]. Trastuzumab and pertuzumab have complementary mechanisms of action and potential synergist effects on antitumor activity when administered together [38,39].

### 5.3. Antibody–Drug Conjugate

Ado-trastuzumab emtansine (T-DM1) was the foremost antibody–drug conjugate (ADC) targeting HER2 to be approved by the FDA [40,41] (Figure 1). T-DM1 is composed of the monoclonal antibody trastuzumab linked to a microtubule polymerization inhibitor, mertansine (DM1), via a non-cleavable thioether linker [42,43].

### 5.4. TKIs

The tyrosine kinase inhibitors (TKIs) lapatinib and neratinib, through competitive molecular mechanisms, target the catalytic kinase in the HER2 intracellular domain, promoting a disruption of the ATP phosphorylation-dependent processes, which leads to cancer cell angiogenesis disruption and tumor shrinkage. While lapatinib is a reversible TKI on the EGFR and HER2 domains, neratinib is an irreversible TKI targeting the HER1, HER2, and HER4 pathways [8,44].

## 6. Defining Risk Categories

While achieving pCR is the most valuable predictive factor associated with better recurrence-related results, pathologic residual disease (pRD) indicates a higher risk for the poorest survival and for disease relapse [45]. At baseline, HER2+ BC patients with tumors > 20 mm and with the detection of LN+ prior to surgery, or postoperatively (following NAC with trastuzumab), and with residual tumors > 10 mm or LN+ are highly associated with early relapse events [45,46,47]. When there is pRD in the breast only, tumors > 10 mm have an estimated increased recurrence risk of 16% when compared to sub-centimeter tumor sizes or pCR. When pRD is seen in LNs, there is an increased relapse risk by at least 50% in those with ≥2 LNs+ when compared to those with 1 LN+ [46].

Other contributory factors related to increased risk and linked to worse recurrence rates are an age less than 50 years at the time of diagnosis, low levels of tumor-infiltrating lymphocytes (TILs), as well as a body mass index (BMI) ≥ 25 [45,48].

The BC recurrence risk attributed to the ER status appears to be linked to the time at which chemotherapy is delivered, whether it is administered before or after surgery, but more supportive data around this are needed. HER2+ BC with ER− is more likely to achieve pCR as compared to the ER+ phenotype, reflecting higher chances of relapse in the latter group [9,15]. However, in BC patients with pRD, many studies did not find a correlation between the ER status and OS or DFS [49,50,51]. Nevertheless, though less prone to attaining a tumor response due to decreased chemotherapy sensitivity, ER+ HER2+ patients who achieve pCR are likely to have a favorable prognosis [45].

**Figure 2:**
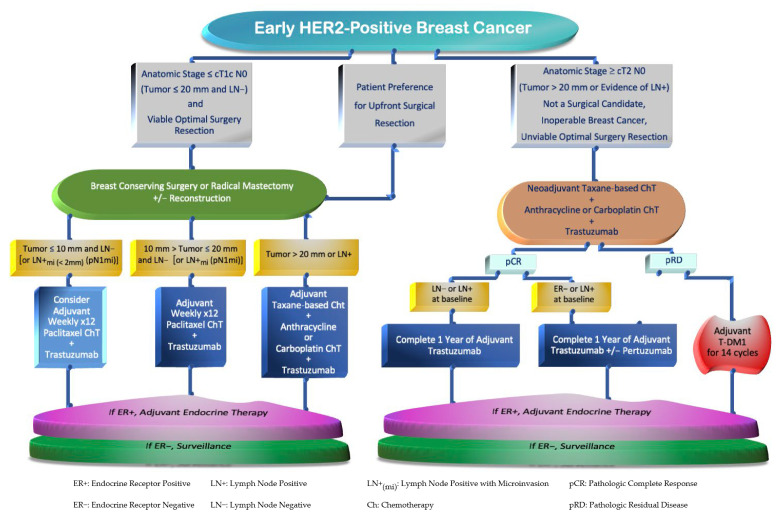
Flowchart of Systemic Therapy Strategies in Early HER2-Positive Breast Cancer.

For physician decision making, it is important to identify which patients will benefit from systemic treatment upfront versus after surgery. These decisions are typically determined by the BC risk of recurrence. The evaluation of factors such as breast cancer staging based on radiological and clinical–pathological features characterizing the breast tumor size, nodal involvement, and HER2 levels of expression is required [52]. For example, BC with uninvolved LN and tumors measuring up to 20 mm, which correspond to anatomic stages (by The American Joint Committee on Cancer—AJCC [53]) I to IIA, are preferred for upfront surgery, followed by adjuvant systemic treatment [54]. Conversely, high-risk patients with larger tumors (≥T2 stage or >20 mm) and/or with axillary LN+ involvement, encompassing BC stages IIB and III, are mostly suitable for a multimodal disciplinary approach which consists of the following: neoadjuvant systemic therapy, followed by radical or conservative breast surgical resections, usually along with LN assessment with sentinel LN biopsy or axillary LN dissection [54,55]. These approaches should be followed by adjuvant systemic treatment and, if appropriate, the subsequent administration of endocrine and local radiation therapies [52]. The selection between HER2 blockers to be given after surgery would depend on microscopic pathology appraisal, where patients with pRD on the surgical specimen should be considered for adjuvant treatment modalities [9] (Figure 2).

## 7. Adjuvant Treatment

### 7.1. Very Low-Risk HER2+

Most randomized controlled trials (RCTs) for early HER2+ BC excluded patients with tumors ≤ 10 mm with no LN involvement (T1abN0) [56]. The data available for this population are mostly derived from non-randomized studies with a reduced sample size in most cases [36,57]. Hence, it is not clear whether small HER2+ tumors should be treated with standard-of-care single-agent chemotherapy and trastuzumab. A recent systematic review and meta-analysis, including 12 studies and almost 7000 patients, investigated whether patients with T1abN0 HER2+ BC would benefit from adjuvant trastuzumab. A significant DFS improvement was seen in patients treated with versus without chemotherapy and trastuzumab. The absolute increased benefit of trastuzumab over no trastuzumab was 7% at a 5-year DFS and less than 1% at the 3-year OS. However, even in those patients who did not receive trastuzumab or chemotherapy, their prognosis was reasonably good, with a 5-year OS of 95.9% and a DFS of 88.3% [58]. Only a few studies evaluated the effect of chemotherapy plus trastuzumab in small HER2+ tumors based on the ER status, suggesting that a larger population sample is needed to ascertain robust conclusions regarding these subgroups [58,59]

### 7.2. Low-Risk HER2+

A meta-analysis that assembled data from five trials and included 4220 patients with breast tumors ≤ 20 mm (pT1c) evaluated participants who were randomized to receive adjuvant trastuzumab versus no trastuzumab. With a median follow up of 8 years, there was a substantial OS and DFS advantage for those treated with trastuzumab. However, the compiled population in this meta-analysis was highly heterogeneous, as it included patients with LN– and LN+ as well as different lengths of trastuzumab administration varying from 9 weeks to 24 months [60].

The phase II APT trial was a single-arm study that recruited HER2+ BC patients with LN– and tumors < 3 cm in size, although 91.1% of that study population had tumors ≤ 2 cm. The patients received 12 administrations of weekly paclitaxel with trastuzumab, followed by one year of maintenance trastuzumab [61,62]. The updated 7-year analysis showed an OS of 95% (95% CI, 92.4–97.7) and a DFS of 93.3% (95% CI, 90.4–96.2%) [62]. The 10-year breast cancer-specific survival (BCSS) was 98.8% (95% CI, 97.6–100%) [63]. Interestingly, despite the omission of anthracycline chemotherapy in the APT trial, the long-term benefit continues to be seen in low-risk HER2+ BC patients [62].

The ATEMP trial is a phase II study that, with a 3:1 allocation ratio, compared the efficacy between 1 year (or 17 cycles) of T-DM1 versus paclitaxel with trastuzumab, the TH regimen for HER2+ BC with stage I disease, in the adjuvant setting. The study result showed a significant 3-year iDFS advantage of 4.4% on T-DM1 [64]. Although there was no difference in the quality of life (QOL) assessment between the two arms, higher rates of treatment discontinuation and dose reductions were seen in patients treated with T-DM1 versus TH, and these were more frequent in patients with an age above 50 years. Hence, T-DM1 could potentially be an alternative option for patients who prefer to avoid alopecia and neuropathy, both of which are typical adverse events (AEs) induced by taxane chemotherapy [64,65].

In summary, very low-risk and low-risk HER2+ BC, defined as anatomic stage T1ab and T1cN0, respectively, with a clinical or radiological breast tumor size estimation of 20 mm or less and with no suspicious LN or biopsy-proven LN at baseline, are candidates for receiving 12 cycles of adjuvant paclitaxel chemotherapy and trastuzumab for 1 year, as long as the surgical findings are consistent with the pre-surgical evaluation.

### 7.3. High-Risk HER2+

In the higher-risk groups comprising BC stages II or III, the combined analysis of the phase III trials NSABP B-31 and NCCTG N9831 showed that, regardless of the breast tumor size, ER status, LN involvement, or age groups, trastuzumab had a substantial OS and DFS benefit in all subgroups [15,29]. In these trials, women with HER2+ BC, with LN+ or LN–, and with high-risk features (defined as ER+ with a tumor size > 20 mm and ER− with tumors > 10 mm) were randomly allocated to receive adjuvant chemotherapy with doxorubicin and cyclophosphamide, followed by weekly paclitaxel (AC→T), plus/minus 1 year of trastuzumab. At 8.4 years, those who received trastuzumab had a significant risk reduction in mortality of 37% (95% CI, 0.54–0.73) and a reduction in relapse events of 40% (95% CI, 0.53–0.68) [29].

It is a viable option to deliver systemic therapy in the adjuvant setting for HER2+ BC patients with high-risk features, with clinical or radiological staging ≥ T2 or pathological evidence of LN+. However, this high-risk population would potentially benefit more by receiving treatment in a neoadjuvant scenario. This is because up to 65% of them could achieve pCR post-NAC and hence derive additional long-term survival benefits [11,12].

### 7.4. Adjuvant Dual HER2 Blockage

The first phase III RCT to use the dual HER2 blockage trastuzumab and pertuzumab (H+P), in combination with taxane-based chemotherapy, was the CLEOPATRA trial, which included patients with metastatic or unresectable HER2+ BC. This study showed a survival extension of 16.3 months with the dual combination when compared to trastuzumab alone [66]. In an attempt to replicate these results in the adjuvant scenario, the phase III, double-blinded randomized APHINITY trial tested trastuzumab plus/minus pertuzumab in patients with LN+ or high-risk LN– HER2+ BC [67]. There was no significant OS advantage between trastuzumab alone and that in combination with pertuzumab, with HR 0.83 (95% CI, 0.68–1.02). The updated 8-year OS was 92.7% for dual combination therapy H+P and 92.0%, for single therapy. There was a relative IDFS improvement in reducing distant and locoregional recurrence of 23% (95% CI, 0.66–0.91). In the analysis of subgroups, there was an absolute IDFS benefit of 4.5% (95% CI, 0.59–0.87) in patients with LN+ and one of 3% (95% CI, 0.59–0.92) in those with ER− [68].

The phase III KAITLIN trial recruited HER2+ BC patients with a high risk, with 89.8% of the population having LN+. The participants were randomly assigned to receive an adjuvant anthracycline-containing regimen followed by taxane chemotherapy in combination with H+P versus anthracycline followed by pertuzumab with 18 cycles of T-DM1. The combination H+P was administered for 1 year. There was no invasive DFS difference between the two study arms [69].

None of the adjuvant trials showed a significant OS advantage by using single versus dual HER2 blockage. However, the usage of adjuvant H+P demonstrated a significant but small advantage in select HER2+ BC patients, indicating that those with LN+ and ER− benefit the most from dual combination therapy.

## 8. Neoadjuvant Treatment

Candidates who derive the greatest advantage from receiving upfront systemic therapy and trastuzumab are those with high-risk features in HER2+ BC, patients with inoperable diseases, or those with an unfeasible optimal surgical resection at baseline.

In the randomized phase III NOAH trial, women with HER2+ BC with locally advanced or inflammatory disease were assigned to receive NAC alone versus NAC with trastuzumab followed by 1 year of maintenance trastuzumab. At 5 years, the absolute difference in EFS was 15% (HR 0.64 (95% CI, 0.44–0.93)), and in BCSS, it was 13% (HR 0.59 (95% CI, 0.37–0.92)). These outcomes favored those treated with NAC containing trastuzumab. In these trials, there was a high correlation between pCR and enhanced EFS in patients who received trastuzumab [70].

Knowing that pCR is a potential surrogate marker associated with improved survival outcomes, a systematic review and meta-analysis of seventy-eight RCTs, with 25,150 HER2+ BC patients treated with NAC, showed a significant association between pCR and enhanced OS. The OS at 3 and 5 years revealed a significant mortality rate reduction in those who had pCR with a respective HR of 0.25 (95% CI, 0.13–0.47) and 0.26 (95% CI, 0.20–0.33) [13]. When compared to patients who had pRD, the 5-year absolute OS advantage in those who reached pCR was 13.5%, and the event-free survival was 19.6%. These results highlighted the importance of pCR as a prognostic and surrogate factor for longer survival. Although, over time, as the survival differences amplify in those who had pCR versus pRD, a precise estimate of the survival advantage remains difficult to quantify [13,14]. Notably, many trials explored whether adding a second HER2 blocker to trastuzumab in the neoadjuvant setting would maximize the treatment response, further highlighting the importance of optimizing pCR rates in HER2+ BC.

The NeoSphere is a phase II, open-label randomized trial comparing the addition of pertuzumab to standard trastuzumab with the neoadjuvant FEC→D regimen, which comprises 5-fluorouracil, epirubicin, and cyclophosphamide followed by docetaxel (FEC→DH+P) versus standard-of-care NAC with trastuzumab (FEC→DH). The NeoSphere’s result showed a pCR improvement rate of 16.8% in the arm containing pertuzumab. Notably, almost one-third of the NeoSphere study population reached pCR, and this outcome was identified in 45.8% (95% CI, 36.1–55.7) of patients treated with H+P and in 29% (95% CI, 20.6–38.5) of those who received the single-mAB trastuzumab [71]. The intervention group with dual-HER2 blockage (H+P) was associated with higher 5-year PFS rates (86% versus 81%, respectively (HR 0.69 (95% CI, 0.34–1.40))) [71,72]. In the long term, there were no issues from a safety profile standpoint with the addition of pertuzumab. The left ventricular ejection fraction (LVEF) dysfunction was found to be 3% in the H+P combination group, compared to 0% in the trastuzumab group [71,72].

Another meta-analysis of twenty-six studies which included 9,872 patients evaluated the safety and efficacy of the dual HER+ blockers. AEs such as rash, diarrhea, epistaxis, mucosal inflammation, and anemia were significantly more frequent with H+P as compared to with trastuzumab alone, whereas myalgia was less frequent (OR 0.91 (95% CI, 0.82–1.01); *p* = 0.072) in the combination treatment. Nevertheless, these AEs were deemed acceptable and manageable. Moreover, no significant difference in cardiac toxicity was observed between these therapies (OR 1.26 (95% CI, 0.81–1.95)) [73]. Regarding the efficacy endpoint, the study revealed that NAC with H+P significantly increased the pCR rate (OR 1.33 (95% CI, 1.08–1.63)). The ER− subgroup had the most substantial pCR outcome from the combined H+P treatment versus trastuzumab alone, with an absolute pCR rate almost three times higher, when compared to patients with ER+ status in HER2+ BC [73].

In the phase II KRISTINE trial, 444 patients were randomized to receive six cycles of neoadjuvant T-DM1 plus pertuzumab (T-DM1+P) versus docetaxel, carboplatin, and trastuzumab plus pertuzumab (TCH+P). In the adjuvant setting, patients previously allocated to each of the study arms received 12 cycles of T-DM1+P or H+P, based on randomization [74]. The pCR rates were reached in 44.4% of the study participants in the T-DM1+P and in 55.7% in the TCH+P groups. Thus, there was an absolute advantage in achieving pCR of 11.3% ((95% CI, 20.5–2.0); *p* = 0.016) in the TCH+P group versus the control group. Relapse events (HR 2.61 (95% CI, 1.36–4.98)) were more frequent in patients treated with T-DM1+P, including locoregional progression before surgery (6.7% versus 0%). Treatment discontinuation due to AEs was four times higher in the TDM1-containing arm as compared to the group receiving TCH+P [74,75].

A meta-analysis involving four phase II–III RTCs (CALGB-40601, ChER−LOB, NSABP-B41, NeoALTTO) included 1410 HER2+ BC patients treated with NAC and trastuzumab, lapatinib, or both combined. The pooled analysis showed that lapatinib plus trastuzumab, compared to trastuzumab alone, significantly improves OS and recurrence-free survival (RFS) by reducing the relative risk of mortality and recurrence by 35% and 37%, respectively. In the subgroup of ER− HER+ patients, pCR was correlated with a 73% decreased risk of mortality [76]. With more frequent grade ≥ 3 AEs and higher rates of early treatment discontinuation in the lapatinib-containing arms (predominantly diarrhea and rash), the role of lapatinib in early HER2+ BC has been dubious [77,78,79].

## 9. Adjuvant HER2 Therapy in Postoperative Pathologic Residual Disease

Knowing that patients who did not achieve pCR have worse BC outcomes, the phase III KATHERINE trial attempted to improve relapse rates in HER2+ BC patients who had pRD on the surgical specimen following taxane-based NAC (with or without anthracycline) plus trastuzumab. Notably, almost 20% of the accrued study population received dual HER2+ monoclonal antibodies in the neoadjuvant setting. The participants were assigned to receive 14 cycles of T-DM1 or trastuzumab, postoperatively. At 3 years, 88.3% of patients treated with T-DM1 and 77.0% of those treated with trastuzumab were free of recurrence, translating to a 50% risk reduction of combined invasive BC recurrence and death events in the T-DM1 arm versus the control arm with trastuzumab (HR 0.50 (95% CI, 0.39–0.64)). The OS analysis was premature (HR 0.70 (95% CI, 0.47–1.05)), as a sufficient number of death events were not statistically seen in both study arms [80]. AEs of grade three and of any grade were 5.5% and 10.3% higher, respectively, in the T-DM1 group [51,80].

The ExteNET is a phase III RCT that recruited 2,840 participants with stages I–IIIc HER2+ BC. This study investigated whether extending the adjuvant dual blockage therapy provides a survival advantage. Patients who had received standard-of-care surgery and chemotherapy and had completed adjuvant trastuzumab were enrolled to receive 1 year of neratinib versus placebo [81,82]. The most common AE of neratinib was diarrhea, with 40% being classified as a grade 3 event. The iDFS at 5 years was 90.2% and 87.7% in the intervention and control groups, respectively. A subgroup analysis of 1,631 ER+ patients revealed a 40% iDFS risk reduction in those treated with neratinib (95% CI, 0.43–0.83) [82]. Among the ER+ group, which had an early initiation of neratinib/placebo within 1 year after the completion of adjuvant trastuzumab, the absolute iDFS improvement was 5.1% (HR 0.58 (95% CI, 0.41–0.82)) [83]. Finally, the 8-year OS analysis showed no benefit with the usage of neratinib as an extended duration of adjuvant HER2 therapy (HR 0.95 (95% CI, 0.75–1.21)). At the 8-year mark, the subgroup analysis of OS revealed no substantial differences between the study arms in the ER+ and ER− groups [84].

## 10. Chemotherapy Backbone with versus without Anthracycline

In terms of chemotherapy, the optimal strategies comprise taxane-based regimens plus/minus an anthracycline component. The Dutch study TRAIN-2, a controlled phase III trial, randomized patients with stage II–III HER2+ BC to receive NAC with or without anthracycline. The anthracycline arm consisted of three cycles of FEC, followed by six cycles of carboplatin with paclitaxel. In the non-anthracycline groups, the patients received six or nine cycles of carboplatin with paclitaxel. Both parties received H+P [85]. At 3 years, the estimated EFS was 92.7% versus 93.6% (HR 0.90 (95% CI, 0.50–1.63)), and the OS was 97.7% versus 98.2% (HR 0.91 (95% CI, 0.35–2.36)) in those who received NAC with versus without anthracycline, respectively. These results revealed that HER2+ BC patients can safely have anthracycline omitted without compromising BC prognosis [85,86]. No statistical difference was seen in the pCR rates between the two groups. In those treated with anthracycline, there was a higher incidence of febrile neutropenia and LVEF impairment (7.7% versus 3.2%; *p* = 0.04), when compared to treatment without anthracycline. In this trial, cardiac dysfunction was defined as a drop of ≥10% in LVEF from baseline or a decrease of <50% of the total LVEF. However, symptoms secondary to cardiac dysfunction were rare in both groups, with 1% versus 0, respectively. Regardless of the ER or LN status, these results support that the omission of anthracycline within NAC can be a safe and effective option and does not compromise a patient’s BC outcomes [85,86].

In the randomized phase III BCIRG 006 trial, HER2+ BC patients were allocated to receive one of the following three adjuvant treatment regimens: AC→T, AC→TH, or TCH. Approximately 72% of the accrued trial participants had LN+, from which a third of the total population had LN+ ≥ 4 axillary nodes. No significant differences in OS or DFS were identified between the two study arms containing trastuzumab that compared anthracycline versus non-anthracycline chemotherapy [35,87].

A recently published systematic review and meta-analysis included 11 RCTs with a total of 1,155 patients comparing NAC with versus without anthracycline in the HER2+ BC population. There was no difference in pCR rates between those comparators (OR 0.95 (95% CI, 0.61–1.48)). Amid these two arms, there was no difference in the rates of breast-conserving surgery (OR 1.18 (95% CI, 0.93–1.49)). However, a critical point to note in this meta-analysis was the heterogeneity of the population, comprising patients with a broad range of risk factors that could have potentially affected the study results [88].

The phase II TRYPHAENA trial, which was primarily focused on assessing cardiac safety, included 225 women with HER2+ BC who were to receive NAC with dual HER2 mAB blockage. Patients who were allocated to the anthracycline group received the FEC→DH+P regimen. The anthracycline sparring group comprised six cycles of TCH+P [89]. NAC promoted similar pCR event rates and recurrence outcomes of DFS and PFS between the study groups. And those who were exposed to anthracyclines were more likely to develop LVEF dysfunction when compared to patients exposed to the anthracycline-free regimen [89,90].

Hence, in high-risk HER2+ BC populations, doublet platinum taxane-based chemotherapy regimens, such as carboplatin with docetaxel, have been demonstrated to be a comparable alternative in select patients who should be spared from receiving anthracycline.

## 11. Optimal HER2 Treatment Duration

With regard to the escalation and de-escalation of treatment, the HERA trial did not show a benefit when adjuvant trastuzumab was extended to 2 years, compared to the 1-year standard duration [91]. Three of the most relevant non-inferiority trials with similar study designs, comparators, and endpoints investigated the optimal duration of mABs against HER2, comparing a 6- versus 12-month administration of adjuvant trastuzumab [92]. While the PERSEPHONE study achieved its prespecified non-inferior boundaries for OS and DFS at 4 years (HR 1.07 (95% CI, 0.93–1.24), *p* = 0.011), the PHARE and HORG trials failed to support that the treatment with a shorter schedule is non-inferior to the standard duration at the 7.5-year and 3-year analyses, respectively [93,94,95]. To tiebreak these contrasting results, a high-level evidence meta-analysis compiled RCTs comparing 6 versus 12 months of adjuvant trastuzumab and emphasized that the shorter treatment length conferred a significantly reduced DFS benefit, with an HR of 1.22 ((95% CI, 1.09–1.38); *p* = 0.0008), despite the less frequent cardiotoxicity events [96,97]. A 6-month duration of trastuzumab reflected an increased absolute DFS risk by almost 4% at 5 years [98].

Despite the many attempts to achieve comparable outcomes by abbreviating or stretching the course of trastuzumab, the 1-year duration remains the optimal time for which trastuzumab should be delivered in the adjuvant setting.

## 12. Multigene Profiling Assays

The genomic expression assays (GEAs) help in tailoring optimal treatment whilst also helping to avoid unnecessary chemotherapy in women with BC ER+ HER2− [99]. By means of generating predictive risk scores, GEAs identify patients in whom adjuvant chemotherapy can be advantageous [99,100].

Given similar scores among BC tissue samples from biopsy and surgical specimens, the St. Gallen International Consensus Guidelines endorsed the use of GEAs to optimize neoadjuvant treatment strategies from biopsied samples, which has helped contribute to a practice change [101,102].

For patients with HER2+ BC disease, the novel HER2DX is a prognostic and predictive GEA encompassing 27 gene expression levels including ErbB2 mRNA, which, in combination with clinical and tumor features, identifies four gene signature profiles (immune, proliferation, luminal differentiation, and HER2 amplicon) [103]. The HER2DX is a validated gene profiling assay that can estimate the BC recurrence risk and NAC tumor response, quantifying the likelihood in achieving pCR [104]. The HER2DX can be helpful by determining in whom the escalation or de-escalation of chemotherapy is the most favorable strategy [18,103].

## 13. Tumor-Infiltrating Lymphocytes

Antitumor immune response can lead to the recruitment and formation of well-organized clusters of T-lymphocytes (mainly, CD4+T and CD8+T), B-lymphocytes, as well as natural-killer (NK) cells. In other words, TILs are such lymphocytic cells that move towards, and surround, the microenvironment of tumor cells [105,106]. Quantifying the volume of TILs embodying the breast tumor is essential in determining TIL levels [107,108]. The greater the TIL index, the higher the ratio between CD8+ and CD4+ T cells, implying an enhanced cytotoxic T-cell reaction [109]. In these cases, a high number of TILs has been associated with a better NAC response and improved outcomes in HER2+ and TNBC, in addition to being a reliable histologic predictor in determining HER2 positivity when IHC results are equivocal (score 2+) [107,110,111].

A recent meta-analysis included 29 published trials and 9145 participants with BC treated with NAC. In this pooled-analysis, a high number of TILs were demonstrated to be predictive of pCR (OR 2.54 (95% CI, 1.50–4.29)) and prognostic for OS (HR 0.93 (95% CI, 0.87–0.99)) and DFS (HR 0.96 (95% CI, 0.94–0.98)) in HER2+ patients. However, no association was found in ER+ BC patients [112]. In another study, the secondary analysis of the NeoALTTO trial, which included patients treated with neoadjuvant lapatinib and trastuzumab, demonstrated that for each 1% increase in the level of TILs, there was a 3% decrease in the BC relapse event rate [113].

TIF can also be predictive of a better efficacy of immune checkpoint inhibitors (ICIs) in BC patients [114]. Together with TILs, the programmed death-1 (PD-1) ligand-1 (PD-L1), as immune biomarkers, can identify tumors that are sensitive to immunotherapy, helping clinicians to select potentially successful candidates to be treated with ICIs [110].

## 14. Immune Checkpoint Inhibitors

By blocking signaling transduction on HER2-enriched cells with targeted HER2 blockers, an immune-mediated response is triggered. Thus, HER2 blockers can be interpreted through the lens of an immunotherapeutic strategy [115]. Emerging data have demonstrated potential synergism in promoting antitumor immunity between HER2 therapies and cytotoxic T lymphocyte-associated antigen 4 (CTLA4) or PD-1/PD-L1 inhibitors in BC patients [116,117]. In the metastatic setting, the PANACEA trial showed a durable clinical benefit of pembrolizumab with trastuzumab in heavily treated HER2+ BC patients with PD-L1+ tumors [118]. On the other hand, the KATE2 study did not show an advantage of adding atezolizumab to T-DM1 in previously treated advanced HER2+ BC [119].

The phase III IMpassion050 trial recruited high-risk (T2–4; N1–3) early HER2+ BC patients. Participants were randomized to receive atezolizumab/placebo with standard anthracycline-taxane based NAC with H+P. After surgery, trial candidates continued H+P and atezolizumab/placebo until a 1-year completion of the mABs against the HER2. This study showed that those with PD-L1-positive tumors had higher pCR rates versus those with PD-L1-negative tumors. No substantial difference in pCR rates was noted when atezolizumab was added to standard chemotherapy [120].

To date, TNBC has been the only subtype among all BC phenotypes to show a substantial benefit from being exposed to the combination NAC and ICI, leading to a practice change [121,122]. Ongoing trials continue to explore a potential role for checkpoint inhibitors in the management of early HER2+ BC. The ASTEFANIA trial is evaluating adjuvant atezolizumab/placebo in combination with T-DM1 in patients with pRD after surgery [123]. The APTneo is investigating NAC with atezolizumab with H+P in BC patients with high-risk features [124]. The NeoHIP is a neoadjuvant trial accruing candidates with a high risk for BC relapse, comprising weekly paclitaxel, with H+P with versus without pembrolizumab. After surgery, the selection of systemic treatment is in accordance with their treating physician’s discretion [125].

## 15. Future directions

### 15.1. Novel Targeted Therapies and Innovative Strategies

The antibody–drug conjugate trastuzumab deruxtecan (T-DXd) is a humanized mAB against the HER2 pathways, entwined with a topoisomerase-I inhibitor payload along with a tetrapeptide-centered cleavable linker. T-DXd has shown efficacy not only in the overenhanced HER marker but also within a range of low-HER expressions in HER2+ BC [126]. The positive results of the DESTINY-Breast03 trial demonstrated durable antitumor activity and an outstanding 67% risk reduction PFS with T-DXd versus T-DM1 in metastatic HER2+ disease. These results led to questions about whether T-DXd would be a promising adjuvant treatment in patients with operable HER2+ BC disease [127]. To answer this and other queries, the DESTINY-Breast05 is continuing investigation as an ongoing phase III, randomized, active-controlled trial of T-DXd versus T-DM1 in HER2+ BC patients, with high-risk features for disease relapse, who have residual invasive disease in the breast and/or axilla after NAC completion [128]. The DESTINY-Breast11 is also a phase III trial recruiting patients with high-risk HER2+ disease, comparing T-DXd followed by paclitaxel with H+P versus standard-of-care AC→TH+P [129].

Tucatinib is a reversible TKI that selectively targets EGFR-HER2 domains in HER2 enhanced cell lines [130]. Through ATP-competitive mechanisms, tucatinib inhibits phosphorylation and blocks the proliferation of downstream HER2 signaling [131]. The HER2CLIMB trial tested the addition of oral tucatinib with capecitabine and trastuzumab in metastatic HER2+ BC and showed an increased OS and PFS in patients previously treated with H+P and T-DM1 [7,132]. Due to its small molecule, tucatinib has the ability to cross the blood–brain barrier, providing sustainable control in patients with active brain metastasis [133]. The CompassHER2 Trial is a phase III, randomized study investigating the superiority of T-DM1 with versus without tucatinib in high-risk HER2+ BC patients who had pRD after neoadjuvant treatment with HER2–directed therapy [134].

Margetuximab is an engineered modified chimeric immunoglobulin G1 (IgG1) mAB that had undergone structural modification on the IgG1 crystallizable fragment (Fc) region [135,136]. Aiming to enhance innate and adaptive immune responses against HER2, margetuximab was designed to overenhance the affinity between IgG1-Fc and CD16A and their variants relative to the IgG1 wild type [137,138]. With the aim of identifying a better drug than trastuzumab, the phase III SOPHIA trial tested margetuximab with chemotherapy versus trastuzumab with chemotherapy in metastatic HER2+ BC patients who were previously heavily treated [136]. Although margetuximab achieved the desired safety endpoints, the final analysis failed to show an OS advantage of margetuximab versus trastuzumab [136,139]. However, the SOPHIA trial showed that there is a potential survival improvement of margetuximab in selected patients harboring CD16A allelic variants [139].

Another RCT evaluating margetuximab is the MARGOT study. This is an ongoing phase II trial recruiting HER2+ BC patients with anatomic stage II–III to compare the combination paclitaxel and pertuzumab with either margetuximab or trastuzumab in the neoadjuvant setting [140].

Pyrotinib is an oral TKI that, with irreversible mechanisms, targets the HER1, HER2, and HER4 domains [141]. Pyrotinib plus capecitabine has met PFS and intracranial objective response rate (ORR) endpoints, with acceptable AEs in patients with advanced HER2+ BC [142,143]. Based on the PHOEBE and PERMEATE trials, pyrotinib has been approved in China to be administered with capecitabine following progression on pertuzumab and trastuzumab in the metastatic setting [142,143,144]. With the intent to replicate the benefit of pyrotinib in the early BC setting, the PHEDRA trial is a randomized controlled study recruiting patients with high-risk HER2+ BC, a tumor size ≥ 2 cm, and LN+. The participants are assigned to receive neoadjuvant FEC→DH, in addition to daily pyrotinib versus placebo. After the completion of NAC, the subsequent cancer therapy is selected at the physicians’ discretion following current and local guidelines. The endpoints of pCR and ORR were higher in the pyrotinib group by 19.0% (95% CI, 9.5–28.4) and 9.7% (95% CI, 2.7–16.6), respectively, compared to the control group [144]. The OS data had not yet been reached in this study. The most common AEs of pyrotinib are grade three or four diarrhea, arising in more than 44% of cases [144,145].

Ongoing RCTs include NAC with pyrotinib, pertuzumab, trastuzumab, and nab-paclitaxel for HER2+ BC [146]. Therapies compounded between ADC and TKIs are being explored, such as the ARX788, which is an anti-HER2 agent with a tubulin payload. The ARX788 with pyrotinib is being compared to a doublet platinum chemotherapy such as the TCH+P in the neoadjuvant scenario [147].

### 15.2. De-Escalation of Adjuvant Therapy

Aiming to identify the undefined boundaries between low- and high-risk HER2+ BC, pCR has been utilized as a prognostic tool and a surrogate marker to determine the de-escalation of adjuvant cytotoxic treatments [148]. The DECRESCENDO trial was designed to assess the efficacy of the de-escalation of adjuvant anti-HER2 therapy following pCR [149,150]. This is a dual-phase single-arm phase II trial evaluating the neoadjuvant single-agent taxane chemotherapy combined with subcutaneous (SC) applications of trastuzumab and pertuzumab. However, prior to undergoing neoadjuvant treatment, the trial candidates were required to have breast tumor sizes between 1.5 and 5.0 cm and LN uninvolved [151]. After surgery, participants who achieved pCR on breast and axillary specimens received adjuvant pertuzumab and trastuzumab via SC application for an additional 14 cycles. In those with pRD, in addition to low to intermediate levels of residual cancer burden (RCB), 14 cycles of adjuvant T-DM1 were given, and those with more substantial RCB received four cycles of AC chemotherapy. The primary analysis of the DECRESCENDO trial showed an overall pCR rate of 56.7%. Efficacy endpoints regarding the de-escalation of adjuvant therapy are still ongoing [149,150].

With an identical design and similar eligibility criteria, the DAPHNe and CompassHER2 pCR trials appraised postoperative trastuzumab and pertuzumab with the omission of adjuvant chemotherapy in HER2+ BC patients. While the phase II, CompassHER2 pCR accrued patients with stage II–IIIA, the phase I pilot study DAPHNe included participants with a tumor size ≥ 1.5 cm and stage II–III, but with the exception of inflammatory or T4d disease, as these were exclusionary conditions. In both studies, prior to trial enrolment, all candidates received taxane single chemotherapy with H+P. In the adjuvant setting, candidates who had pCR were assigned to H+P, without any further chemotherapy [149,152,153].

Following a similar intent of sparing patients from receiving chemotherapy, the phase II PHERGain trial was presented at the American Society of Clinical Oncology (ASCO) 2023 international conference. This study explored de-escalating adjuvant chemotherapy based on a pathological response-adapted approach using ^18^F-FDG-PET. The PHERGain recruited women with stage I–IIIA HER2+ BC with PET-evaluable and surgically operable breast tumors ≥ 1.5 cm. Participants were evaluated for an early response by PET, which corresponded to a tumor size reduction of ≥40% from baseline [154]. Participants in the chemotherapy-free arm received dual HER2 blockers with H+P for two cycles, after which nearly 80% had PET evidence of an early response. These patients, who continued on doublet mABs for an additional six cycles until surgery, had a pCR rate of 37.7% on surgical specimens. Postoperatively, patients who had pCR continued H+P for 10 additional cycles, and those who had pRD were allocated to receive 6 cycles of adjuvant chemotherapy consisting of the TCH+P regimen, followed by H+P and ET, if applicable. Conversely, patients who did not reach the desired PET response after the first two cycles of H+P following randomization were allocated to receive six cycles of TCH+P until surgery and subsequently received adjuvant H+P and ET, if ER+ [155]. The analysis of the intention-to-treat (ITT) population revealed a 3-year iDFS of 95.4% ((95% CI, 92.8–98); *p* < 0.001) seen in the group where chemotherapy was omitted. Among these, patients who were considered PET responders, and hence did not receive chemotherapy, had a 3-year iDFS rate of 98.8% (95% CI, 96.3–100.0) [155].

All of these studies, exploring the de-escalation of adjuvant chemotherapy, will potentially help identify which patients are the best candidates in whom omitting adjuvant chemotherapy is appropriate.

### 15.3. Other Oncodriver Mutations and Molecular Pathways Crossing with HER2+

#### PI3K/AKT/mTOR/MAPK Pathways

The phosphatidylinositol 3-kinase (PI3K) is a group of heterodimeric enzymes encoded by catalytic subunits of the PIK3CA gene [156]. The PIK3 protein kinase B (AKT), aligned with the mammalian target of rapamycin (mTOR), plays an essential role in conducting information to the intracellular domain that can lead to cell differentiation, proliferation, and apoptosis, as well as DNA repair [156,157]. Targeted therapies against HER2 act by interrupting signal conduction through the PI3K/AKT/mTOR and MAP kinase (MAPK) pathways, suppressing cellular regulation mechanisms [158,159].

Everolimus is an oral selective mTOR inhibitor approved in metastatic ER+ HER2– BC [160]. The combination of everolimus and trastuzumab was shown to have synergism by reducing tumor growth in HER2 overexpression [161]. Nevertheless, PIK3CA mutations have been associated with tumorigenesis and acquired resistance to anti-HER2 therapy [162,163]. In HER2+ BC, PIK3CA mutation has been considered predictive of a poor treatment response due to lower pCR rates and decreased DFS, as compared to wild-type PIK3CA [164,165]. Similarly, a higher risk of disease recurrence was seen in patients with metastatic HER2+ BC harboring PIK3CA gene alteration in the EMILIA and CLEOPATRA trials, despite the dual HER2 blockage [166,167].

Alpelisib is a selective PI3K inhibitor capable of inhibiting cell proliferation by suppressing signaling through both wild-type and mutated PI3K-alpha pathways [168,169]. Although alpelisib did not show survival benefits when given in ER+ HER2– BC, its usage with trastuzumab is being tested in the ALPHABET trial in advanced HER2+ BC and could provide insights about their adjunct effect in patients with PI3KCA mutations [170].

To date, none of the PI3K inhibitors have been tested in early HER2+ BC. However, harboring PIK3CA mutations is predictive of worse survival outcomes when compared to those without this mutation [171]. Thus, a better understanding of the mechanisms for evading resistance to the HER2-targeted therapies through the PI3K/AKT/mTOR/MAPK pathways is needed to improve outcomes in BC patients with PIK3CA mutations [159].

### 15.4. Cancer-Targeted Vaccines

Breast cancer is a heterogeneous disease with immunogenic pathogenesis. The HER2 molecular pathway plays multiple roles, including one as an oncogenic driver [19,172]. Research highlights the importance of understanding the conceivable association between ErbB2-specific immunity mechanisms and acquired treatment resistance from targeted HER2 treatments [173]. The ErbB2-antigen is targetable with vaccines that potentially trigger high levels of antibodies, which, through the mediation of lymphocyte T cells, elicits the suppression of the Th-1 response and T-cell cytotoxic proliferation in overexpressed tumor-antigens [174]. Following this approach, numerous clinical trials aim to induce anti-tumor effects with a specific HER2–directed T-cell response by using peptide-based vaccines or viral vectors and self-replicating RNA-based cancer vaccines administered alone or in conjunction with immune checkpoint inhibitor anti-PD1 mABs [19,175,176]. Although the induction of immunity by anti-cancer vaccines appears to have a crucial role in suppressing tumor activity, the magnitude and duration of their treatment effect have not yielded breakthrough results to change the treatment course of HER+ BC [175,177].

Another immunomodulatory strategy targeting tumor cells with a tumor antigen-specific antibody involves tailored-fusion proteins. This approach, designed to bind to the cell surface of the HER2 antigen, has demonstrated some biological activity in conjunction with HER2 treatments towards inhibiting the tumor growth of HER2–enriched cells [178,179]. Targeting fusion proteins may have a supplemental role, especially when administered with vaccines against the HER2 antigen to elicit a boost of immune responses in cancers where HER2 is overexpressed [179,180].

## 16. Conclusions

This literature review highlights the latest evidence around early HER2+ BC (Table 1). In summary, categorizing patients into appropriate BC risk groups, by way of evaluating the tumor and LN staging, is decisive in guiding clinicians towards the neoadjuvant versus adjuvant route. In patients with low-risk disease with tumors measuring up to 20 mm and LN–, there are sufficient data to support the omission of anthracycline, and in this case, upfront surgery, followed by adjuvant paclitaxel with trastuzumab, is the preferred choice. In the high-risk category comprising larger tumors or in cases of axillary lymph node involvement, the neoadjuvant approach to downsizing tumors would be the first option. Notably, systemic treatment with versus without anthracycline chemotherapy seems to have comparable results, as there is no efficacy-related difference among them. Hence, doublet platinum-based chemotherapy using carboplatin with taxane is a reasonable alternative for those in whom anthracycline is contraindicated. It is important to emphasize that, post-NAC, evidence of pCR is predictive and a surrogate marker of better survival outcomes, but in those with residual disease, adjuvant T-DM1 is the standard indication for reducing BC recurrence rates.

Evidence supports that neoadjuvant treatment with the dual HER2 blockage trastuzumab and pertuzumab promotes superior pCR and PFS outcomes, when compared to single-agent trastuzumab. However, OS data are limited or non-existent around the dual combination, and the association between enhancing pCR rates and the long-term clinical advantage with the addition of pertuzumab remains unknown [181]. Nevertheless, adjuvant trastuzumab and pertuzumab uphold a significant but small recurrence reduction advantage in select HER2+ BC patients. In this case, those with LN+ and ER− benefit the most from dual combination therapy.

If there are funding restrictions or limitations in accessing the combination trastuzumab and pertuzumab in the neoadjuvant or adjuvant settings, the single HER2 mAB trastuzumab continues to be a very reasonable option.

There was a role for adjuvant neratinib in high-risk groups of ER+ HER2+ BC patients who completed adjuvant trastuzumab, but the overall recommendation in recent trials suggests the benefit is small, especially when taking into consideration treatment with the combination of pertuzumab and trastuzumab, or with T-DM1 for residual disease, all of which are less toxic and more tolerable than neratinib.

Regarding the optimal treatment duration, one year of adjuvant trastuzumab conferred an extensive survival advantage and remains the standard therapy length for early HER2+ BC. Nevertheless, shorter schedules of trastuzumab may be considered as an alternative for patients with borderline cardiac conditions or in those with a lower risk of BC relapse.

Topical areas of the current discussion, with respect to evaluating the de-escalation of adjuvant chemotherapy, include ongoing trials with forthcoming results that could help point towards patients in whom eliminating further chemotherapy is advantageous.

In this manner, tailoring the breadth of HER2+ treatment strategies hinges on a handful of factors for consideration in each individual case. Bearing in mind the patients’ personal preferences among the suitable options and medical history and identifying the BC risk category are critical in supporting optimal treatment decisions.

## Figures and Tables

**Table 1:** Summary of key clinical trials, systematic reviews, and meta-analyses that investigated the role of HER2 therapy in HER2-positive breast cancer.

Study	Interval	Recurrence Outcome (95% CI)	Survival Outcome (95% CI)
Low-Risk HER2+			
**Hassing et al.** (2023) [58], a meta-analysis of 12 trials (n = 6927) Setting: adjuvant. Population: T1abN0 Treatment: 1. Trastuzumab and Cht 2. No trastuzumab ± Cht	5 years	Group 1 vs. 2 DFS, HR 0.14 (0.07–0.29); *p* < 0.0001 Group 1: DFS of 95.3% (92.7–97.9) Group 2: DFS of 88.3% (84.8–91.8)	Group 1 vs. 2 OS, HR 0.17 (0.04–0.66); *p* = 0.011 Group 1: OS of 98.5% (97.2–99.8) Group 2: OS of 95.9% (94.2–97.6)
**O’Sullivan et al.** (2015) [60], a meta-analysis of five trials (n = 2263) Setting: adjuvant. Population *: T1 N0–1 Treatment: taxane-based Cht with vs. without trastuzumab * 87% of patients had LN = 0	8 years	ER+: DFS, HR 0.70 (0.59–0.85); *p* < 0.001 ER−: DFS, HR 0.66 (0.49–0.88); *p* < 0.001 Recurrence rate: ER+: 17.3% vs. 24.3%; *p* < 0.001 ER−: 24.0% vs. 33.4%; *p* < 0.001	ER+: OS, HR 0.68 (0.52–0.89); *p* = 0.006 ER−: OS, HR 0.59 (0.47–0.74); *p* < 0.001 Mortality rate: ER+: 7.8% vs. 11.6%; *p* = 0.005 ER−: 12.4% vs. 21.2%; *p* < 0.001
**APT** (2019) [62], a phase II, single-arm trial (n = 406) Setting: adjuvant. Population: T1–T2 (≤3 cm) N0 Treatment: TH (trastuzumab weekly paclitaxel × 12)	7 years	DFS of 93.3% (90.4–96.2%)	OS of 95% (92.4–97.7)
**ATEMPT** (2021) [64], a phase III trial (n = 497) Setting: adjuvant. Population: T1abcN0 Treatment: 17 cycles of T-DM1 vs. TH (weekly paclitaxel × 12 and trastuzumab for 1 year)	3 years	iDFS of 97.8% vs. 93.4%; *p* < 0.0001. RFI of 99.2% (98.2–100) vs. 94.3% (89.2–98.8)	–
High-Risk HER2+			
Joint analysis of the phase III **NSABP B-31** and **NCCTG N983** trials, Perez et al. (2014) [29] (n = 4046) Setting: adjuvant. Population: any LN+, ER+ ≥ T2 N0, or ER− ≥ T1cN0 Treatment: AC→T with vs. without trastuzumab	10 years	DFS, HR 0.60 (0.53–0.68); *p* < 0.001 DFS of 73.7% vs. 62.2% ER+: DFS of 76.1% vs. 65.1% ER−: DFS of 70.9% vs. 58.6%	OS, HR 0.63 (0.54–0.73); *p* < 0.001 OS of 84% vs. 75.2% ER+: OS of 86% vs. 77.1% ER−: OS of 81.6% vs. 73%
**NOAH** (2014) [70], a phase III trial (n = 235) Setting: neoadjuvant. Population: stage II–III LN+ Treatment: anthracycline-based Cht with vs. without trastuzumab	5 years	EFS, HR 0.65 (0.45–0.95); *p* = 0.024 EFS of 58% (48–66) vs. 43% (34–52)	OS, HR 0.68 (0.44–1.05); *p* = 0.083 OS of 74% (64–81) vs. 63% (53–71)
Dual Anti-HER2 blockage			
**APHINITY** (2017) [67], (2022) [68], a phase III trial (n = 4805) Setting: adjuvant. Population: T1–3 LN+ or LN– and high-risk (T > 2 cm) Treatment: anthracycline-based Cht with H+P vs. trastuzumab alone	6 years 8 years	LN+: IDFS, HR 0.72 (0.59–0.87) IDFS, HR 0.77 (0.66–0.91) IDFS of 88.4% vs. 85.8%	– OS, HR 0.83 (0.68–1.02); *p* = 0.078 OS of 92.7% vs. 92.0%
**KAITLIN** (2022) [69], a phase III trial (n = 1846) Setting: adjuvant. Population *: LN+, ER+: T1–3 or ER-: T2–3 Treatment: AC→TH+P vs. AC→TDM1+ P * 89.8% of the population had LN+	3 years	IDFS of 94.2% vs. 93.1% LN+: HR 0.97 (0.71–1.32; *p* = 0.83)	–
**NeoSphere** (2012) [72], a phase II trial (n = 417) Setting: neoadjuvant. Population: T2–3 N0–3 or T4a-c LN+ or LN– Treatment *: Group A: TH. Group B: TH+P. Group C: H+P. Group D: TP * Patients received FEC + trastuzumab in an adjuvant setting	5 years	PFS, group A: 81% (72–88), group B: 84% (72 –91), group C: 80% (70–86), and group D: 75% (64–83) All groups combined: pCR vs. pRD: PFS of 85% vs. 76%, HR 0.54 (0.29–1.00)	–
**KRISTINE** (2018) [74] (2019) [75], a phase II trial (n = 444) Setting: neoadjuvant and adjuvant. Population: stage II–III, (T > 2 cm) Treatment: (N)T-DM1+P → (A)T-DM1+P vs. (N)TCH+P → (A)H+P	3 years	pCR rate of 44.4% vs. 55.7%; *p* = 0.016 EFS, HR 2.61 (1.36–4.98) EFS of 85.3% vs. 94.2%	–
**Guarneri et al.** (2022) [76], a meta-analysis of four trials (n = 1410) Setting: neoadjuvant. Population: stage I–III Treatment: Cht and trastuzumab, lapatinib, or both combined	6–7 years	H+L: RFS, HR 0.62 (0.46–0.85) All groups combined: pCR sub-group: RFS, HR 0.45 (0.34–0.60)	H+L: OS, HR 0.65 (0.43–0.98) All groups combined: pCR sub-group: OS, HR 0.32 (0.22–0.48)
Adjuvant HER2 therapy in postoperative pathologic residual disease			
**KATHERINE** (2019) [80], a phase III trial (n = 1486) Setting: adjuvant. Population: stages I–IIIc (except T1aN0 or T1bN0). All patients had pRD and received taxane-based NAC * with HER2 therapy Treatment: TDM-1 versus trastuzumab * 76.9% of patients received Cht with anthracycline; 18.3% received H+P in a neoadjuvant setting	3 years	DFS, HR 0.50 (0.39–0.64); *p* < 0.001	OS, HR 0.70 (0.47–1.05); *p* = 0.08
**ExteNET** (2021) [83] (2022) [84], phase III trial (n = 2840) Setting: adjuvant. Population: stages I–IIIc Treatment: after the completion of Cht and trastuzumab, the patients received adjuvant neratinib for 1 year or placebo	5 years 5 and 8 years	iDFS, HR 0.60 (0.33–1.07) iDFS of 90.2% vs. 87.7% − At 5 years:DDFS, HR 0.57 (0.39–0.83) DDFS of 92.4% vs. 87.7% (84.8–90.1)	− At 8 years: OS, HR 0.95 (0.75–1.21) OS, ER+ group: HR 0.80 (0.58–1.11) OS, ER− group: HR 1.18 (0.83–1.69)
Chemotherapy with vs. without Anthracycline			
**TRAIN-2** (2018) [85], a phase III trial (n = 408) Setting: neoadjuvant. Population: stage II–III Treatment: Cht, trastuzumab, and pertuzumab with vs. without anthracycline	3 years	PCR rate of 67% vs. 68%; *p* = 0.95 EFS, HR 0.90 (0.50–1.63) EFS of 92.7% vs. 93.6%,	OS, HR 0.91 (0.35–2.36) OS of 97.7% vs. 98.2%
**BCIRG 006** (2011) [35] (2016) [87], a phase III trial (n = 3222) Setting: adjuvant. Population *: T1–3 LN+ or high-risk with LN– Treatment: group A: AC→T, group B: AC→TH and group C: TCH * 72% of patients had LN+	10 years	DFS, group B: AC→TH HR 0.70, (0.60–0.83); *p* < 0.001 DFS, group C: TCH HR 0.76 (0.65–0.90); *p* < 0.001	OS, group B: AC→TH HR 0.64 (0.52–0.79); *p* < 0.001 OS group C: TCH HR 0.76 (0.62–0.93); *p* = 0.0081
**Zhu et al.** (2023) [88], a meta-analysis of 11 trials (n = 1998) Setting: neoadjuvant. Population: operable breast cancer Treatment: Cht, trastuzumab ± pertuzumab with vs. without anthracycline	–	pCR OR 0.95 (0.61–1.48); *p* = 0.83	–

Cht: chemotherapy; DFS: disease-free survival; iDFS: invasive disease-free survival; RFI: recurrence-free interval; EFS: event free-survival; LN: lymph node; pCR: pathologic complete response; pRD: pathologic residual disease; AC→T: doxorubicin and cyclophosphamide, followed by weekly paclitaxel; TP: taxane + pertuzumab; H+P: trastuzumab and pertuzumab; H+L: trastuzumab and lapatinib; T-DM1+P: T-DM1 and pertuzumab; TCH+P: docetaxel, carboplatin, trastuzumab, and pertuzumab; NAC: neoadjuvant chemotherapy; (N): neoadjuvant; (A): adjuvant.
